# O-GlcNAcylated FTO promotes m6A modification of SOX4 to enhance MDS/AML cell proliferation

**DOI:** 10.1186/s12964-025-02058-6

**Published:** 2025-01-23

**Authors:** Junjie Gou, Jingjing Bi, Kexin Wang, Lei Lei, Yanli Feng, Zengqi Tan, Jiaojiao Gao, Yanan Song, Enci Kang, Feng Guan, Xiang Li

**Affiliations:** 1 Key Laboratory of Resource Biology and Biotechnology in Western China, Ministry of Education, Provincial Key Laboratory of Biotechnology, College of Life Sciences, Northwest University, Xi’an, P. R. China; 2 Department of Hematology, Provincial People’s Hospital, Xi’an, P. R. China; 3 Institute of Hematology, School of Medicine, Northwest University, Xi’an, P. R. China; 4 Xi’an Gaoxin No.1 High School, Xi’an, Shaanxi China; 5 College of Life Sciences, Northwest University, 229 Taibai North Road, Xi’an, Shaanxi 710069 P. R. China

**Keywords:** Myelodysplastic syndromes (MDS), Acute myeloid leukemia (AML), O-linked N-acetyglucosamine (O-GlcNAc), Fat mass and obesity-associated protein (FTO), SRY related high mobility group box (SOX4)

## Abstract

Fat mass and obesity-associated protein (FTO) was the first m6A demethylase identified, which is responsible for eliminating m6A modifications in target RNAs. While it is well-established that numerous cytosolic and nuclear proteins undergo O-GlcNAcylation, the possibility of FTO being O-GlcNAcylated and its functional implications remain unclear. This study found that a negative correlation between FTO expression and O-GlcNAcylation in patients with myelodysplastic syndrome (MDS) and acute myeloid leukemia (AML). The decreased O-GlcNAcylation on FTO can result in diminished m6A modification of SRY-related high mobility group box 4 (SOX4). This led to the promotion of cell apoptosis and inhibition of cell proliferation in MDS/AML. The O-GlcNAcylation of FTO stabilized SOX4 transcripts in an m6A-dependent manner, resulting in increased AKT and MAPK phosphorylation and decreased cell apoptosis. Inhibiting FTO O-GlcNAcylation significantly slowed AML progression in vitro, a finding supported by clinical data in MDS/AML patients. In conclusion, our study highlights the crucial role of FTO O-GlcNAcylation in RNA m6A methylation and the progression of MDS/AML, thereby providing a potential therapeutic avenue for these formidable diseases.

**Supplementary Information:**

The online version contains supplementary material available at 10.1186/s12964-025-02058-6.

## Introduction

The m6A modification, also known as N6-methyladenosine, is one of the most prevalent and abundant modifications found in RNA molecules in eukaryotic cells [1]. It involves the addition of a methyl group to the sixth nitrogen atom of adenosine residues within RNA molecules [2]. This modification plays crucial roles in various aspects of RNA metabolism and function, including mRNA stability, splicing, transport, and translation efficiency [3–5]. Additionally, m6A modification has been implicated in diverse biological processes such as cell differentiation, embryonic development, and stress responses [6, 7]. More and more emerging evidence suggested m6A has been defined as a crucial modulator in the progression of hematological malignances including myelodysplastic syndromes (MDS) and acute myeloid leukemia (AML) [8, 9]. m6A modification is a dynamic and reversible process, involving m6A methyltransferases (Writers), demethylases (Erasers), and methylation reading proteins (Readers) [10, 11]. Fat mass and obesity-associated protein (FTO) is the first reported RNA N6-methyladenosine (m6A) demethylase in eukaryotic cells [12]. Since the initial classification of FTO as an m6A demethylase, various studies started to unravel a connection between FTO’s demethylase activity and the susceptibility in tumorigenesis on the molecular level.

Post-translational modifications (PTMs) are covalent chemical modifications occurring on protein amino acid side chains and are dynamic processes with temporal and spatial specificity [13]. Phosphorylation, acetylation, ubiquitination, glycosylation, methylation, SUMOylation and others are among the most widely studied PTMs [14]. Several studies have reported that FTO proteins can be subject to phosphorylation, ubiquitination and SUMOylation [15–17].

As one of the important post-translational modifications, O-linked β-N-acetylglucosamine (O-GlcNAc) modification is a dynamic and reversible event catalyzed coordinately by O-GlcNAc transferase (OGT) or glycoside hydrolase O-GlcNAcase (OGA). OGT, which catalyses the transfer of a GlcNAc moiety from the donor substrate UDP-GlcNAc to the hydroxyl groups of target serine (Ser) or threonine (Thr) residues; and OGA, which catalyzes the hydrolysis of this sugar modification [18]. O-GlcNAcylation is involved in a wide range of cellular processes, including transcription, translation, signal transduction, protein degradation, and cytoskeletal dynamics. Dysregulation of O-GlcNAcylation has been implicated in various diseases, including diabetes, neurodegenerative disorders, cancer, and cardiovascular diseases. However, it is noteworthy that no reports have surfaced regarding O-GlcNAcylation in relation to FTO.

In this study, we reported the negative relationship of FTO and O-GlcNAcylation in patients with MDS/AML. We found that FTO O-GlcNAcylation was elevated during MDS/AML progression. Low levels of O-GlcNAcylation on FTO reduced m6A modification on SOX4 RNA, leading to a decrease in its relative mRNA expression. This reduction in SOX4 expression hinders the activation of the PI3K-AKT and p44/42 MAPK signaling pathways in MDS/AML cells, thereby promoting increased apoptosis.

## Materials and methods

### Cell lines and cell culture

KG1a, a myeloid leukemia cell line, and SKM1, a cell line derived from MDS that progresses to AML, were grown and propagated as described previously [19]. Briefly, KG1a and SKM1 cells were cultured in RPMI 1640 medium (HyClone; Provo, UT, USA) containing 10% fetal bovine serum (FBS; Biological Industries, Beit Haemek, Israel) at 37 °C in a 5% CO_2_ atmosphere.

### Transfectant construction

The full-length FTO, OGT and OGA genes were cloned from cDNA of KG1a cells. Site-directed mutagenesis was performed using fusion PCR. The wild type and the site-specific mutant FTO were individually inserted into the pLVX-AcGFP-N1 plasmids (Takara; Shiga, Japan). Lentiviral vectors were constructed and packaged into HEK-293T cells using the packaging system, pMD2.G and psPAX2, along with polyethylenimine MAX (PEI, Polysciences Inc.; Warrington, PA, USA). Stable transfectants were established by infecting cells with the lentivirus and selecting them using puromycin (Sigma-Aldrich). All siRNA fragments used in this study were purchased from Beijing Tsingke Biotechnology. For siRNA transfection, we followed the instructions for the HighGene Plus Transfection Reagent (ABclonal Technology, Wuhan, China). Briefly, siRNA at a final concentration of 100 nM was mixed with the transfection reagent and added to the cells. After 48 h, RT-qPCR or western blotting was performed for analysis.

### Patient samples

Mononuclear cells were isolated from bone marrow aspirates of patients with MDS/AML and separated by gradient centrifugation through Ficoll-Hypaque (TBD, Tianjin, China) [20]. CD34^+^ cells were sorted using a CD34 microsphere kit (Miltenyi Biotechnology, Bergisch Gladbach, Germany) [21]. CD34^+^ cells were treated with 20 µM DMSO and 20 µg/mL OSMI-1 (MCE, NJ, USA) for 48 h, respectively. Written informed consent, in accordance with the Declaration of Helsinki, was obtained from all patients. All protocols were reviewed and approved by the research ethics committee of Northwest University.

### Proteomic analysis

Protein (500 µg) was denatured with 8 M urea, reduced with 5 mmol/L DTT (Sigma-Aldrich) for 1 h at room temperature (RT), alkylated with 20 mmol/L IAM (Sigma-Aldrich) for 30 min at RT in the dark, diluted with distilled water to reduce the urea concentration below 2 mol/L, digested with lysylendopeptidase (Wako Puro Chemical; Osaka, Japan) and trypsin (Promega; Madison, WI, USA). The peptide mixture was lyophilized, resuspended in 50 mmol/L NH_4_HCO_3_, and purified using Oasis HLB cartridges. Two-dimensional LC-MS were carried out using an LTQ Orbitrap MS (Thermo Fisher, San Jose, CA, USA). Data was analyzed using Proteome Discover (Thermo Fisher) and MaxQuant software program as described previously [22].

### In vivo mouse experiment

Animal experiments were conducted in strict accordance with the guidelines of the Animal Care and Use Committee of Northwest University. Tissue-specific OGT-KO mice were generated using the Cre-LoxP system by crossing OGT-flox (OGT^fl/fl^) mice (Jackson Laboratory, Bar Harbor, ME, USA) with Mx1-Cre mice (Jackson Laboratory) to produce HSC OGT-KO mice. Cre activation was induced by intraperitoneal injection of 250 µg polyinosinic acid (poly I: C, Sigma-Aldrich), and the mice were humanely euthanized after six weeks. Following euthanasia, mononuclear cells were isolated from the femurs of OGT^fl/fl^ and Mx1-Cre; OGT^fl/fl^ mice and further purified using cKit^+^ magnetic beads (Miltenyi Biotec). Subsequently, the cells were infected with MLL-AF9 lentivirus (kindly provided by Professor Guangyao Kong, Xi’an Jiaotong University) to establish a primary AML cell model.

### In vitro O-GlcNAcylation

In vitro O-GlcNAcylation was performed as described [23]. FTO fused with a 6 × His tag, and OGT fused with a GST tag, were individually overexpressed in *E. coli BL21* (DE3) and purified. His-tagged FTO (10 µg), GST-tagged OGT (20 µg) and 2.5 mM UDP-GlcNAc (donated by Prof. Junqiang Fang, Shandong University, China) were added to a 100 µL reaction buffer (125 mM NaCl, 1 mM EDTA and 20 mM Tris HCl, pH 7.4), and reacted at 37℃ for 6 h. O-GlcNAc-modified FTO underwent SDS-PAGE separation and a series of treatments, including dehydration, reduction, alkylation, and trypsin digestion. The resulting peptides were separated using a PepMap C18 nano column on the ThermoFisher U3000 RSLCnano system (#QE HF-X, ThermoFisher, MA, USA). The O-GlcNAcylation sites were analyzed using pFind software [24].

### Western blotting

The cells were collected, rinsed three times with cold PBS and lysed in RIPA buffer containing 50mM Tris (pH 7.2), 1% Triton X-100, 0.5% deoxycholate, 0.1% SDS, 150 mM NaCl, 10 mM MgCl_2_ and 2.5% glycerol. Protein lysates were incubated on ice for 30 min and centrifuged at 14,000 g for 15 min at 4 °C. The supernatant was collected and assayed using BCA kit (Beyotime Biotechnology, Shanghai, China). Proteins were separated by SDS-PAGE and transferred to polyvinylidene difluoride (PVDF) membranes (Merck KGaA, Darmstadt, Germany). The membranes were blocked with 3% (w/v) bovine serum albumin (BSA; Sigma-Aldrich) in TBST (20 mmol/L Tris-HCl, 150 mmol/L NaCl, 0.05% Tween 20, pH 8.0) for 1 h at 37 °C. Subsequently, the membranes were incubated with primary antibodies overnight at 4 °C, followed by incubation with the appropriate HRP-conjugated secondary antibodies (Beyotime). The bands were developed using an ECL solution (Vazyme Biotech, Nanjing, China) and photographed using a gel documentation system (Tanon Science & Technology). Bcl2 (15071, CST, Danvers, MA, USA). Anti-GAPDH (G-9545) was obtained from Sigma-Aldrich (St. Louis, MO, USA), Anti-O-GlcNAcylation (PTM BIO, Hangzhou, China) and anti-m6A (ab286164) were from Abcam (Cambridge, MA, USA). Anti-SOX4, Anti-OGT, Anti-OGA and HA-tag were from Abclonal.

### Immunoprecipitation (IP)

KG1a cells were treated with 20 µM Thiamet G (TMG, MCE) and 20 µg/mL OSMI-1 respectively for 48 h. Cells were lysed with lysis buffer (50 mM Tris-HCl, pH 7.4, 1 mM EDTA, 150 mM NaCl, and 1% Triton X-100) containing Protease Inhibitor Cocktail (4693159001, Roche, Switzerland) and Phosphatase Inhibitor (NCM Biotech, Co, Ltd). After centrifugation, the supernatant was incubated with anti-HA antibody (ABclonal) at 4 °C overnight, centrifuged and collected for further use.

### Protein co-immunoprecipitation (Co-IP)

Cell lysates underwent incubation with anti-FTO antibody (Proteintech, Wuhan, China) overnight at 4 °C, followed by treatment with protein A/G agarose (Santa Cruz Biotechnology, TX, USA) for a duration of 12 h at 4 °C. Subsequently, the agarose was subjected to PBS washing, harvested through centrifugation at 2, 000 g for 5 min, and the samples were subjected to boiling in 1 × SDS Sampling Buffer for a period of 10 min prior to Western blotting.

### m6A dot blot

Total RNA was isolated using the Trizol method (ABclonal). The concentration and purity of the isolated RNA were measured with a DS-11 spectrophotometer (DeNovix Inc.). The total RNA was then denatured at 95 °C for 3 min and spotted dropwise onto a positively charged nylon membrane (Beyotime), which had been pre-blocked with 5% skimmed milk. The membrane was incubated overnight at 4 °C with an anti-m6A antibody (Abcam, 1:2,000). On the following day, an HRP-conjugated anti-rabbit IgG secondary antibody (Beyotime) was applied to the membrane and incubated with gentle shaking for 1 h at room temperature. Enhanced chemiluminescence was used for signal development. Methylene blue staining was performed to confirm equal loading of total RNA on the membrane. The dot blot analysis was performed using a total amount of 400 ng for quantitative analysis.

### Cell apoptosis

1 × 10^6^ KG1a cells were harvested, washed twice with cold PBS and suspended in 1 mL binding Buffer (BioLegend, San Diego, CA, USA). Then 5 µL each of Annexin V and 7-AAD (BioLegend) will be added simultaneously to 100 µL of cell suspension. The cell suspension was vortexed and allowed to incubate for 15 min in the dark at room temperature. 400 µL of 1 × binding buffer was then added to each tube and analyzed by flow cytometry.

### Cell proliferation

Cells were treated with 10 mmol/L EdU (GeneCopoeia; CA, USA) for 4 h. Cells were collected and fixed with 4% paraformaldehyde, permeabilized with 0.2% Triton-X100, and individual samples were incubated with reagents containing 2 µL of Cu_2_SO_4_, 0.5 µL of Alexa Fluor 647 azide, 1 µL of 10 × Reaction buffer additive and 96.5 µL of PBS, mixed well and added to the treated cells, and stained for 30 min at room temperature away from light. Flow cytometry was used to detect EdU fluorescence signals.

### Cell cycle

1 × 10^6^ KG1a cells were washed with cold PBS, fixed with 75% cold ethanol and store overnight at 4 °C. After rinsing with cold PBS, resuspend fixed cells with 0.5 mL PBS, add 100 µg/mL RNase A (YEASEN, Shanghai, China) and 50 µg/mL PI (BioLegend), and incubate at 37 °C for 1 h. Flow cytometry is used for fluorescence signal detection.

### Protein stability assay

To test the stability of O-GlcNAc modification on FTO protein, KG1a cells were treated with 20 µM TMG and 20 µg/mL OSMI-1 for 12 h and then added 50 µM cycloheximide (CHX, MCE) to continue the treatment and cells were collected at 0, 3, 6 and 9 h.

### mRNA stability assay

Cells were treated with 5 µg/mL actinomycin D (S8964, Selleck, TX, USA) for 0, 4, 8, and 12 h, and total RNA was extracted with Trizol. mRNA levels were analyzed by real-time quantitative PCR (RT-qPCR). mRNA half-life (t1/2) was calculated using the following formula: t1/2 = In 2/k_decay_ [25].

### RNA immunoprecipitation and sequencing (RIP and RIP-seq)

Cells were lysed with RIP buffer and subjected to freeze-thaw cycles. Following centrifugation, the supernatant was mixed with anti-FTO antibody-conjugated protein A/G magnetic beads and allowed to incubate for 4–6 h. Subsequently, the immunoprecipitated material was eluted, treated with proteinase K, and underwent RNA extraction using TRIzol. The extent of interaction between FTO and target RNA was assessed through RT-qPCR and normalized against the input. In the case of RIP-seq, rRNAs were removed utilizing the NEBNext rRNA Depletion Kit (New England BioLabs). Subsequently, cDNA libraries were generated and sequenced on the Illumina Novaseq™ 6000 platform from LC-BIO Technology Co.

### Methylated RNA immunoprecipitation sequencing (m6A-seq)

m6A-Seq was performed by LC-Bio Technology Co., Ltd. (Hangzhou, China), according to manufacturer’s instructions. KG1a cells were treated with 20 µM DMSO and 20 µg/mL OSMI-1 for 48 h, respectively, and total RNA was purified and subjected to m6A RNA immunoprecipitation. Eluted m6A-containing fragments (IP) and untreated input control fragments were converted to the final cDNA library following strand-specific library preparation using the dUTP method. The average insert size for the paired-end libraries was approximately 100 ± 50 bp. Paired-end sequencing (PE150) was performed on an Illumina Novaseq™6000 platform.

### MeRIP RT-qPCR analysis

Poly(A) mRNA was purified using the Dynabeads mRNA Purification Kit (Invitrogen). 10% of the RNA was retained as an input control. Protein A/G magnetic beads (Beyotime) were pre-washed and incubated with 5 µg anti-m6A antibody (Abcam) or rabbit IgG for 2 h at 4 °C with rotating. After washing of the conjugate beads, the antibody-conjugated beads were conjugated to the purified poly(A) mRNA and incubated overnight at 4 °C. The m6A RNA was then eluted with elution buffer and recovered by ethanol precipitation. Further enrichment was performed by qPCR and m6A enrichment calculated for each sample by normalization [26].

### Data Analysis

Experimental data were statistically analyzed using GraphPad Prism 9.0 software, with experimental results repeated three times. Statistical comparisons between two groups were made using the unpaired t-test. Significant differences in this thesis are labelled as *P < 0.05; **P < 0.01; ***P < 0.001, respectively.

## Results

### O-GlcNAcylation promotes m6A RNA level

**Fig. 1:**
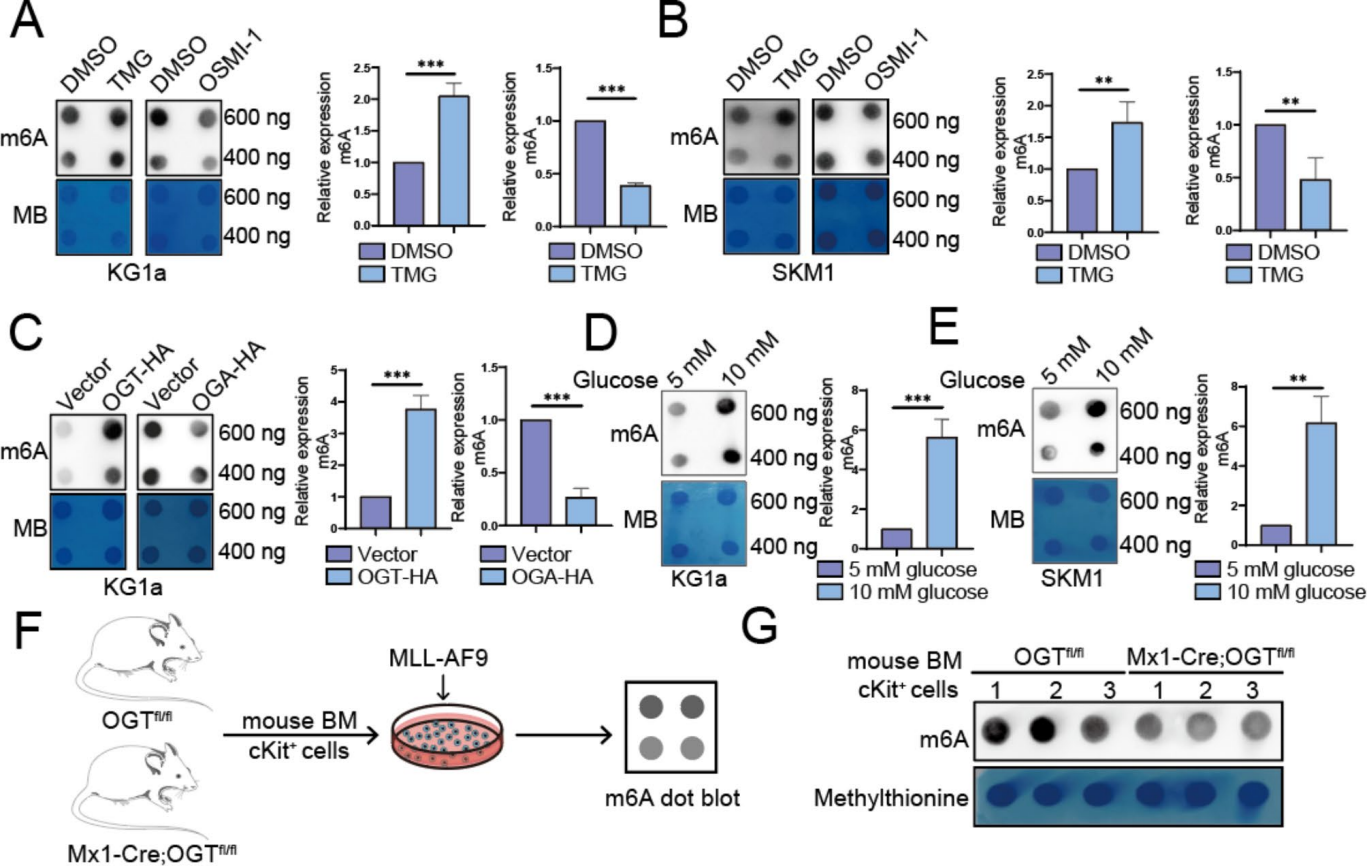
O-GlcNAcylation promotes m6A RNA level. (**A**) Detection of m6A levels after treatment of KG1a cells with 20 µM TMG and 20 µg/mL OSMI-1 by m6A dot blot. (**B**) Detection of m6A levels after treatment of SKM1 cells with 20 µM TMG and 20 µg/mL OSMI-1 by m6A dot blot. (**C**) Effect of transfection of OGT-HA and OGA-HA on m6A level in KG1a cells. (**D**) m6A levels of KG1a cells treated with different concentrations of glucose. (**E**) m6A levels SKM1 cells after treatment with different concentrations of glucose. (**F**) Schematic diagram of bone marrow cKit^+^ cells isolated from OGT^fl/fl^ and Mx1-Cre; OGT^fl/fl^ mice and then transduced with MLL-AF9. (**G**) m6A levels in bone marrow cKit^+^ cells from mice after MLL-AF9 transduction. All experiments described above were independently repeated three times. A total RNA amount of 400 ng was used for quantitative analysis.

Both O-GlcNAcylation and m6A play important roles in the progression of MDS/AML, but the effect of O-GlcNAcylation on m6A level is unknown. As we know, O-GlcNAcylation is controlled by two enzymes: O-GlcNAc transferase (OGT) and O-GlcNAcase (OGA). KG1a and SKM1 cells were treated with the specific O-GlcNAcase inhibitor Thiamet G (TMG) and OGT inhibitor OSMI-1, respectively. It was found that m6A level was increased after TMG treatment, while the opposite result was observed after OSMI-1 treatment (Fig. 1A, B). Furthermore, stable lentiviral transfection of HA-tagged OGT and OGA in KG1a cells showed that m6A levels increased with elevated OGT levels, whereas the opposite effect was observed for OGA (Fig. 1C, S1A). In the glycolytic pathway, about 2–5% of glucose is to produce UDP-N-acetylglucosamine (UDP-GlcNAc) via the branched hexosamine pathway (HBP), which is used for glycosylation modifications on serine or threonine residues of proteins (O-GlcNAc) [18]. Therefore, we tested the effect of altered glucose levels on m6A levels. We found that m6A levels increased in response to high glucose concentrations in KG1a and SKM1 cells (Fig. 1D, E). To further validate the relationship between O-GlcNAcylation and m6A, we isolated bone marrow cKit^+^ cells from OGT^fl/fl^ and Mx1-Cre; OGT^fl/fl^ mice and transduced them with the MLL-AF9 virus to induce leukemia (Fig. 1F). We found cKits^+^ cells from Mx1-Cre; OGT^fl/fl^ mice presented lower m6A levels than those from OGT^fl/fl^ mice (Fig. 1G, S1B). The above results indicate that O-GlcNAcylation increases m6A RNA levels.

### O‑GlcNAcylation weakens FTO expression

**Fig. 2:**
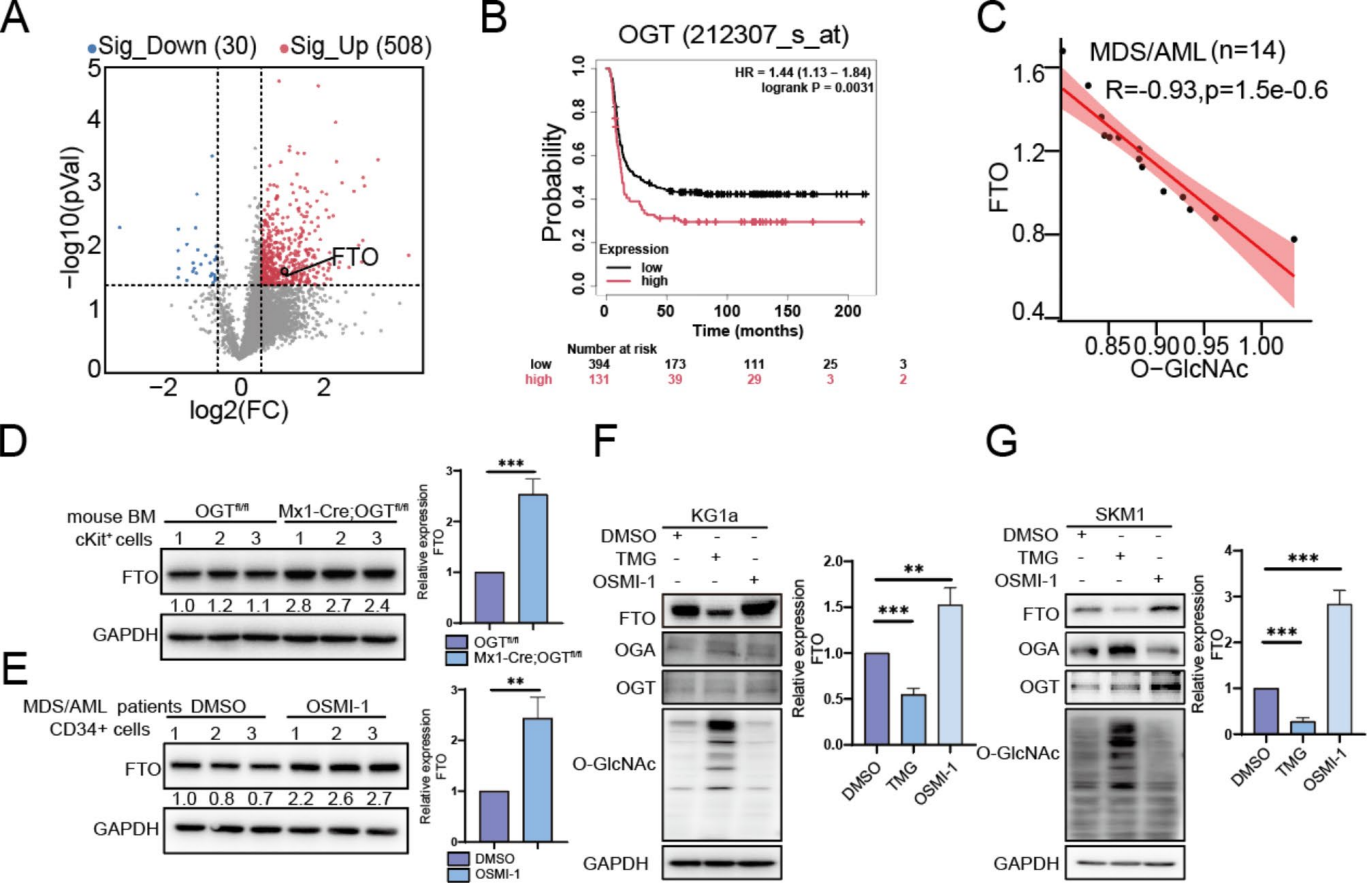
O‑GlcNAcylation weakens FTO expression. (**A**) Volcano diagram representing FTO expression after OSMI-1 treatment in KG1a cells; p-value < 0.05,|Fold change| > 1.5. (**B**) The correlation between O-GlcNAc levels and the survival of AML patients was explored using upper quartile analysis through the Kaplan-Meier Plotter. (**C**) The association between O-GlcNAc and FTO in a cohort of 14 MDS/AML patients by ELISA. (**D**) Western blot detection of FTO level in cKit + cells of the bone marrow from MLL-AF9-induced leukemia mice. (**E**) FTO level in primary CD34 + cells from MDS/AML patients after 20 µM DMSO and 20 µg/mL OSMI-1 treatment. (**F-G**) FTO level in KG1a and SKM1 cells after TMG and OSMI-1 treatment

FTO was the first m6A demethylase to be identified, and FTO undergoes various PTMs including phosphorylation, ubiquitination and SUMOylation [17, 27, 28]. However, whether FTO undergoes O-GlcNAc modification remains unknown. With the stimulation of OSMI-1, FTO expression was increased in KG1a cells profiled by proteomic analysis (Fig. 2A). Interesting, through the Kaplan-Meier Plotter (https://www.kmplot.com), we found that patients with higher OGT levels in acute myeloid leukemia (AML) had a shorter overall survival (OS) (Fig. 2B). Our ELISA results showed that FTO protein expression and O-GlcNAc level in MDS/AML patients are also negatively correlated (Fig. 2C, S2A). Above data indicated that O-GlcNAcylation is negatively associated with FTO expression in MDS/AML.

In the Mx1-Cre; OGT^fl/fl^ mice, we observed the higher FTO expression in cKit^+^ cells, compared to those from OGT^fl/fl^ mice (Fig. 2D). Furthermore, upon treatment with OSMI-1, we noticed an increased level of FTO in primary CD34^+^ cells from MDS/AML patients (Fig. 2E). Consistently, TMG treatment elevated O-GlcNAc levels and reduced FTO expression, while OSMI-1 treatment decreased O-GlcNAc levels and increased FTO expression, in both KG1a and SKM1 cells (Fig. 2F&G).

Similarly, confocal microscopic results showed that FTO expression was reduced after TMG-treated KG1a cells, whereas FTO expression was increased in OSMI-1-treated cells (Fig. S3A). Knockdown of OGA in KG1a cells decreased FTO expression, whereas knockdown of OGT produced the opposite effect (Fig. S3B, C). It was found that high concentrations of glucose increased O-GlcNAc levels in KG1a cells, which decreased FTO expression (Fig. S3D). Taken together, our data suggest that increased O-GlcNAc modification weakens FTO expression.

### O-GlcNAcylation decreases FTO protein stability

**Fig. 3:**
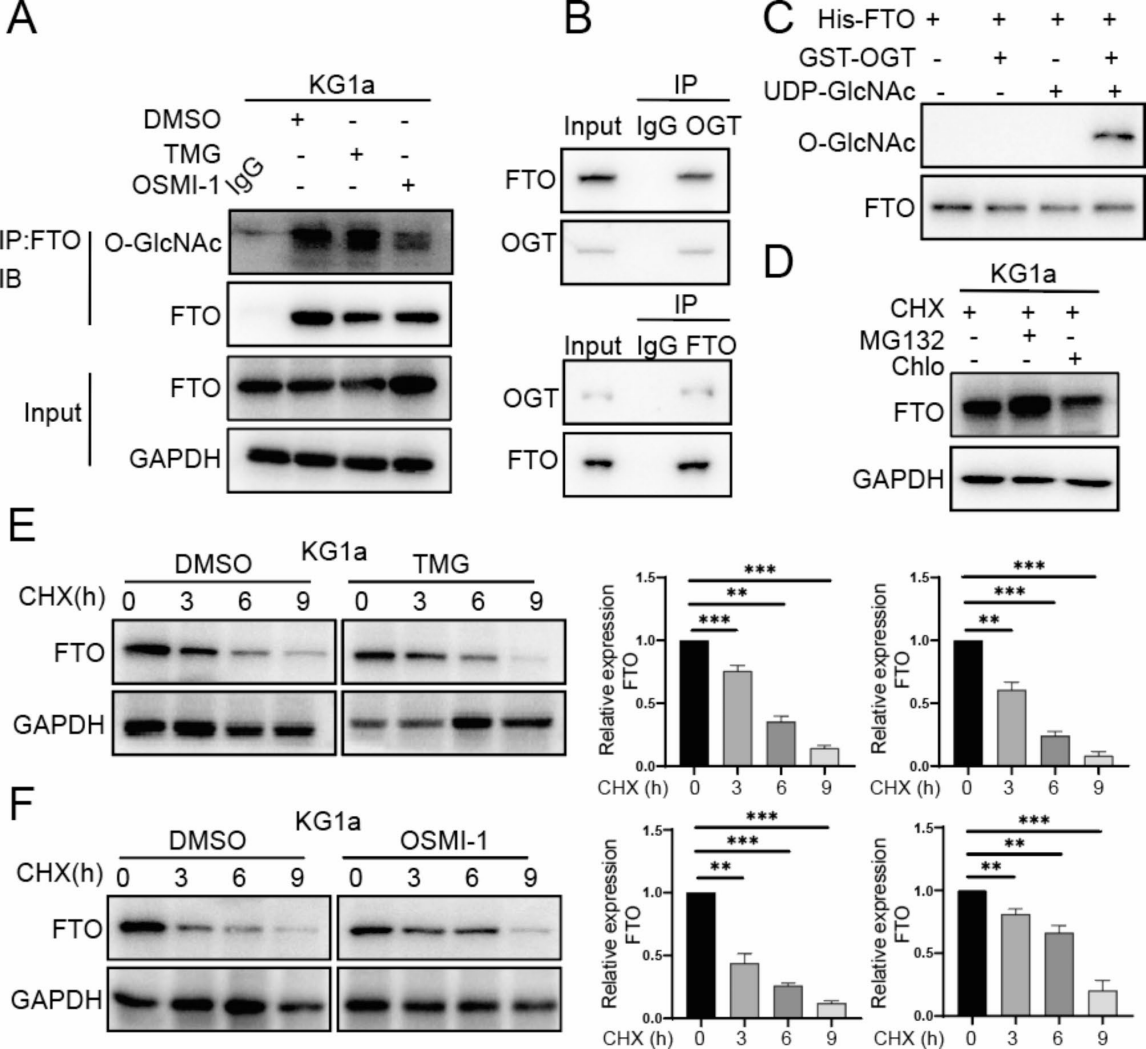
O-GlcNAcylation decreases FTO protein stability. (**A**) O-GlcNAc modification of FTO was identified through IP and Western blotting. Rabbit IgG as negative control. (**B**) Co-IP was carried out to detect the interaction between OGT and FTO in KG1a cells. (**C**) His-FTO was O-GlcNAcylated in vitro and determined by western blotting. (**D**) KG1a was treated with CHX and MG132 or Chlo. FTO degradation was assayed by western blotting. (**E**) FTO expression in KG1a after treatment with TMG and CHX. (**F**) FTO level in KG1a after treatment with OSMI-1 and CHX

Based on the correlation between O-GlcNAc levels and FTO expression, we hypothesize that FTO protein undergoes O-GlcNAc modification. Using immunoprecipitation, we found the presence of O-GlcNAcylation on FTO (Fig. 3A). Co-IP assay also confirmed the interaction between OGT and FTO (Fig. 3B). The in vitro glycosylation model demonstrated that FTO expressed in *E.coli* can undergo O-GlcNAc modification (Fig. 3C). Treatment with TMG or OSMI-1 did not show significant influence on mRNA level of FTO (Fig. S4A), suggesting that O-GlcNAcylation affects FTO expression at post-translational level. When KG1a cells were treated with the protein synthesis inhibitor cycloheximide (CHX) along with the proteasome inhibitor MG132, a notable elevation of FTO expression was observed. However, this increase was not seen with the lysosomal inhibitor chloroquine (Fig. 3D). Furthermore, the increased level of FTO expression appears to correspond with the duration of MG132 treatment (Fig. S4B), indicating that the degradation of FTO primarily takes place through the ubiquitin-proteasome pathway. When treated with CHX, FTO degradation was accelerated under hyper-O-GlcNAcylation conditions (Fig. 3E), but decelerated under hypo-O-GlcNAcylation conditions (Fig. 3F).

### Ser173 is the major O-GlcNAcylation site of FTO

**Fig. 4:**
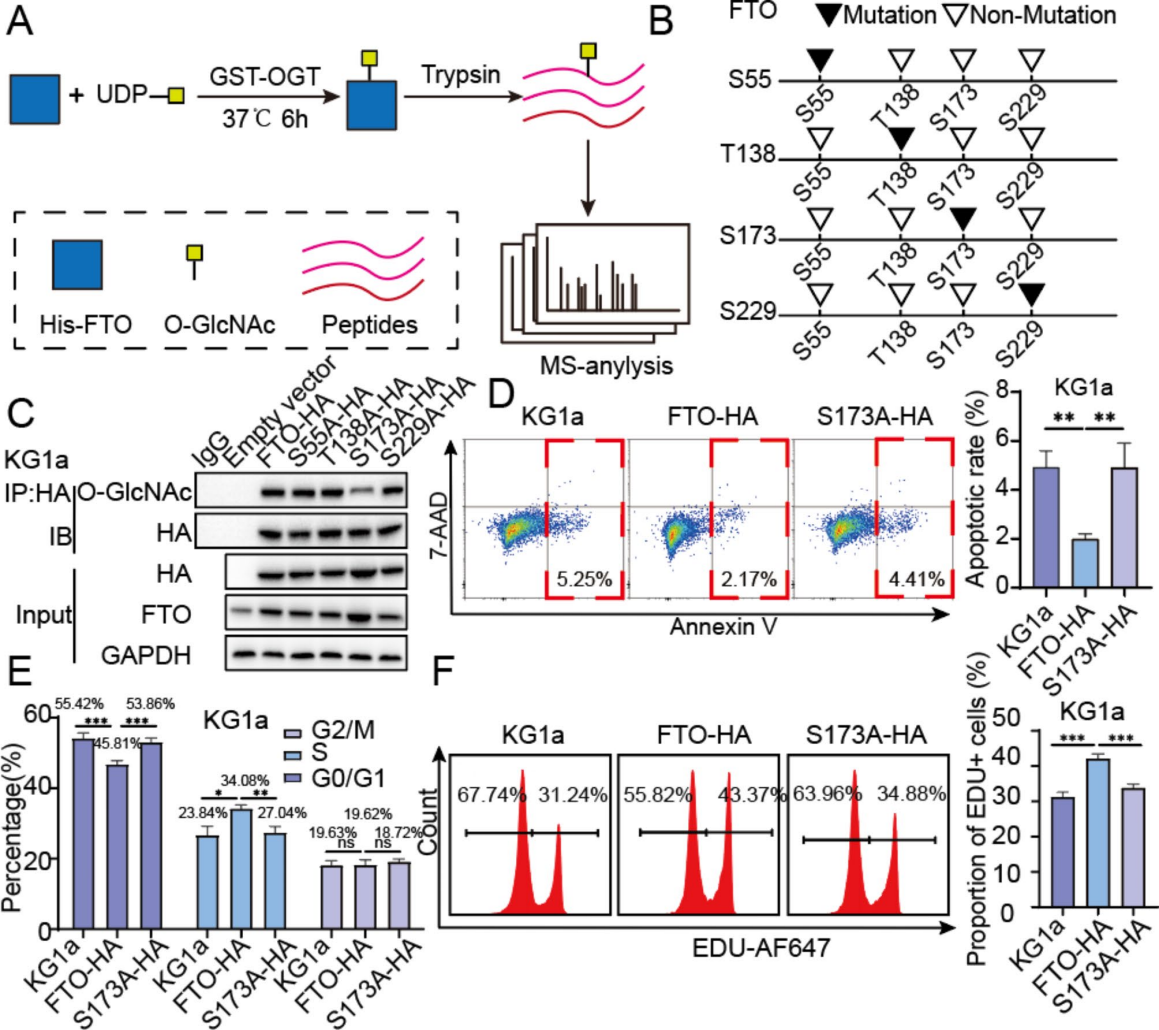
Ser173 is the major O-GlcNAcylation site of FTO. (**A**) Schematic diagram of in vitro O-GlcNAcylation assays. (**B**) Mutation of O-GlcNAcylation sites of FTO. (**C**) Level of O-GlcNAcylation on empty vector control and site-mutated FTO determined by IP and western blotting. (**D**) Cell apoptosis of KG1a, KG1a-FTO-HA, and KG1a-FTO-S173A-HA cells. (**E**) Cell cycle analysis of KG1a, KG1a-FTO-HA, and KG1a-FTO-S173A-HA cells was conducted using flow cytometry. (**F**) Cell proliferation of KG1a, KG1a-FTO-HA, and KG1a-FTO-S173A-HA cells

The O-GlcNAcylated His-FTO was further subjected to mass spectrometry analysis (Fig. 4A). The O-GlcNAcylation at Ser 55, Thr 138, Ser173, and Ser229 were identified (Fig. 4B, S5A). These sites were nutated by replaced with Ala. When expressing the FTO-HA carrying these site mutations (S55A, T138A, S173A, S229A) in KG1a cells, mutations of S173A displayed reduced O-GlcNAc levels, compared to S55A, T138A, and S229A (Fig. 4C). Overexpression of FTO markedly inhibited cell apoptosis, promoted cell proliferation and arrested cells at the G0/G1 phase, relative to KG1a cells. However, the S173A mutation of FTO led to an increase in apoptosis, a decrease in cell proliferation and diminished cell cycle blockage compared to cells overexpressing FTO, bringing the levels back to those observed in non-transfected KG1a cells (Fig. 4D and F). These results indicated that reduced O-GlcNAc modification of FTO was associated with increased apoptosis, cycle arrest at G0/G1 phase concomitant with reduced cell proliferation.

### Identification of FTO targets by high-throughput MeRIP-seq and RIP-seq

**Fig. 5:**
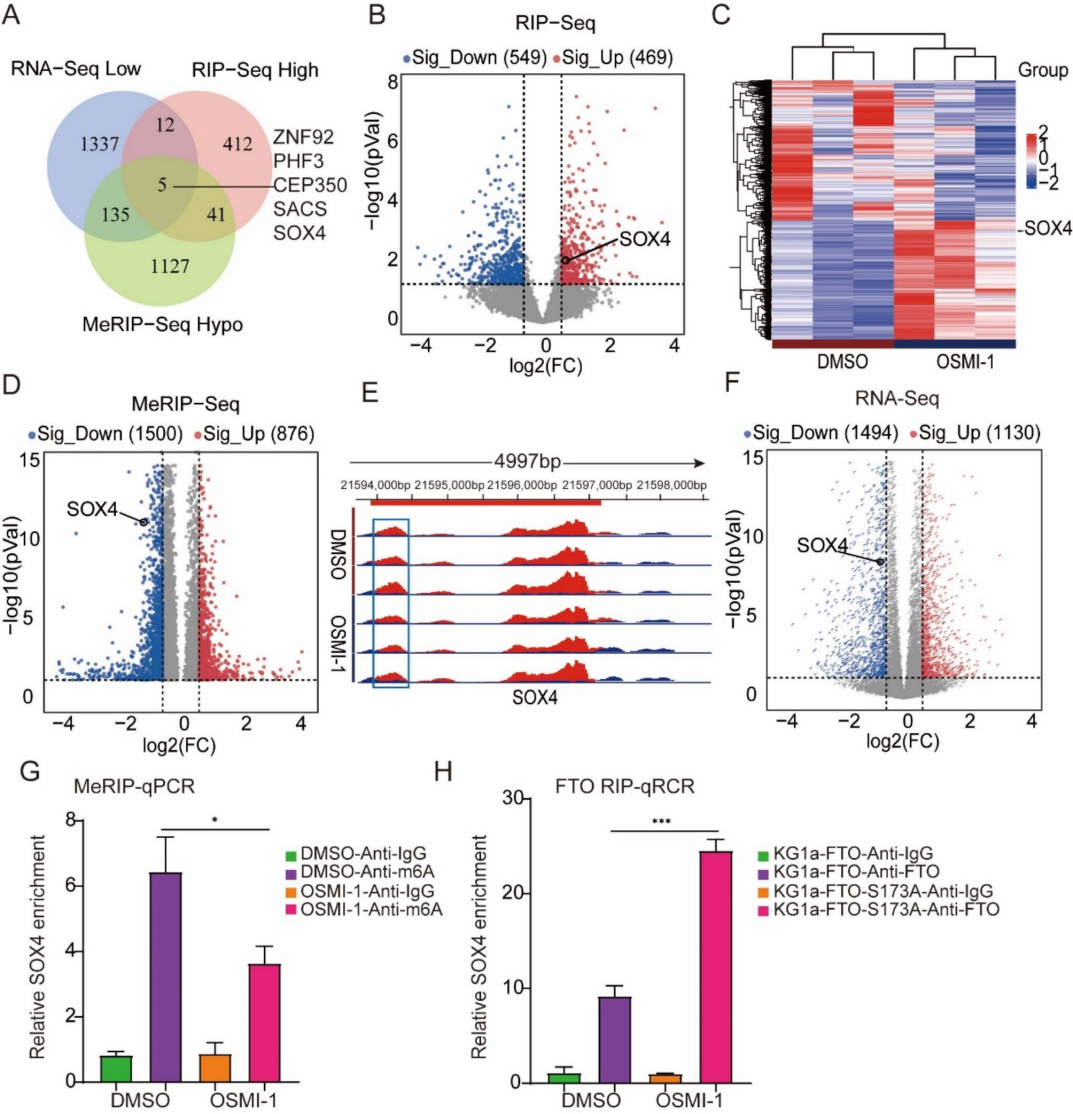
Identification of FTO targets by high-throughput MeRIP-seq and RIP-seq. (**A**) Illustration of overlapping Venn diagrams for RNA-seq (down-regulated genes after OSMI-1 treatment), m6A-seq (down-regulated genes with m6A after OSMI-1 treatment), and RIP-seq (genes with increased binding to FTO after OSMI-1 treatment). (**B**) Volcano diagram showing the genes that bind directly to FTO proteins after the treatment of KG1a cells with OSMI-1. (**C**) Heatmap of KG1a cells treated with OSMI-1 showing genes that bind directly to FTO proteins. (**D**) The volcano diagram of up and down-regulated genes after treatment of KG1a cells with OSMI-1 by m6A sequencing. (**E**) Distribution of m6A peaks across SOX4 transcripts. (**F**) The volcano diagram of up and down-regulated genes after treatment of KG1a cells with OSMI-1 by RNA sequencing. (**G**) MeRIP-qPCR showing SOX4 m6A levels after OSMI-1 treatment of KG1a cells (*n* = 3). (**H**) FTO-RIP-qPCR showing association of SOX4 transcripts with FTO after OSMI-1treatment of KG1a cells (*n* = 3)

FTO was identified as the initial m6A demethylase for eukaryotic mRNA, and its influence on adipogenesis and tumorigenesis is contingent upon its m6A demethylase function [29]. FTO regulates gene expression by removing m6A modifications, which decreases the stability of specific mRNAs. For example, in osteosarcoma, FTO demethylates m6A modifications, reducing the stability of DACT1 mRNA, thereby decreasing DACT1 expression and subsequently activating the Wnt signaling pathway [30]. We speculate that O-GlcNAc modification on FTO may influence the gene expression of m6A modification. By overlapping the data from RNA sequencing (RNA-seq), RNA immunoprecipitation sequencing (RIP-seq), and methylated RNA immunoprecipitation sequencing (MeRIP-seq) with a cutoff of p-value < 0.05 and fold change > 1.5, we identified five m6A-modified genes that bind directly to FTO: ZNF92, PHF3, CEP350, SACS, and SOX4. These genes were upregulated after OSMI-1 treatment and exhibited reduced m6A and RNA levels (Fig. 5A).

Transcription factor SOX4 was identified as a crucial controller of cell differentiation and tumorigenesis [31]. After OSMI-1 treatment, 549 of the genes bound to FTO were significantly down-regulated and 469 were down-regulated, among which SOX4 was one of the up-regulated genes. (Fig. 5B &C). MeRIP-seq analysis of KG1a cells with or without OSMI-1 treatment demonstrated that m6A levels were reduced for 1500 genes and increased for 876 genes. Of these, the m6A level of SOX4 was found to be reduced (Fig. 5D). MeRIP-seq analysis showed that OSMI-1 treatment significantly reduced the m6A level of SOX4 in KG1a cells. (Fig. 5E). Furthermore, analysis of the RNA-seq data indicated down-regulation of the SOX4 gene in KG1a cells post-OSMI-1 treatment (Fig. 5F). To verify the levels of SOX4 m6A after OSMI-1 treatment, a MeRIP-qPCR assay was performed in KG1a cells using anti-m6A (Fig. 5G). The results confirmed that SOX4 m6A levels were reduced by treating KG1a cells with OSMI-1. To further verify that SOX4 is a direct target of FTO, a RIP-qPCR assay was performed in KG1a cells using anti-FTO (Fig. 5H). The results confirmed the interaction between FTO protein and SOX4 mRNA was enhanced after treatment with OSMI-1.

### Reduced O-GlcNAcylated FTO promotes MDS/AML cell apoptosis

**Fig. 6:**
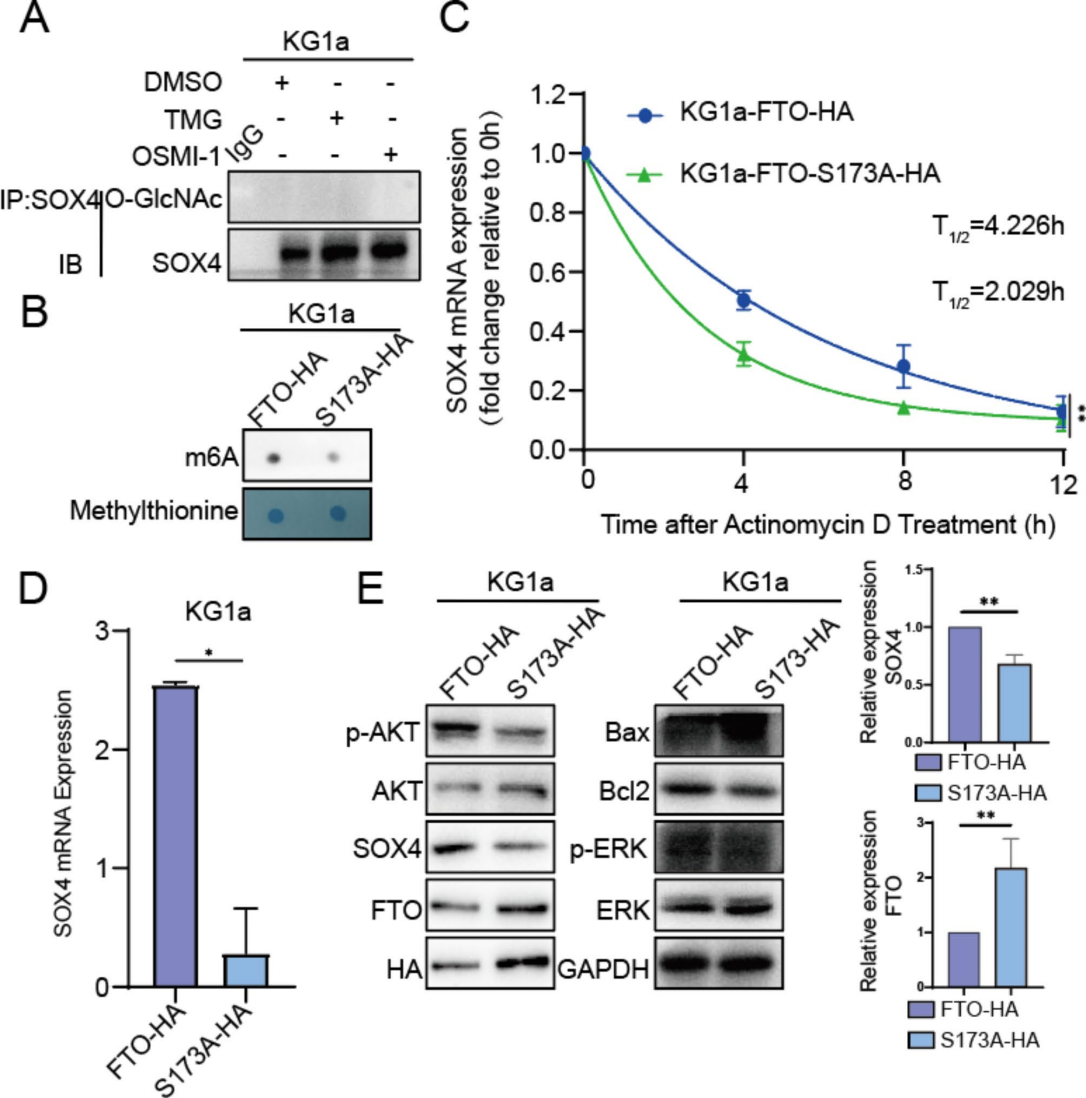
Reduced O-GlcNAcylated FTO promotes the apoptosis of AML cells. (**A**) The O-GlcNAc modification of SOX4 was identified using IP and Western blotting. (**B**) Detection of m6A levels in KG1a-FTO-HA and KG1a-FTO-S173-HA cells. (**C**) Lifetime of SOX4 mRNA in KG1a-FTO-HA and KG1a-FTO-S173A-HA cells. Transcription was inhibited by actinomycin D (5 µg/mL) (*n* = 3). (**D**) Relative mRNA levels of SOX4. (**E**) Detecting FTO, SOX4, p-AKT, p-ERK, Bcl2, and Bax expression in KG1a-FTO-HA and KG1a-FTO-S173-HA cells. This experiment was independently repeated three times

Interestingly, our data showed SOX4 was not modified by O-GlcNAc using IP assay (Fig. 6A). To further investigate the role of SOX4, a downstream target gene of FTO, in disease progression, we used siRNA to silence SOX4 expression in KG1a-FTO overexpression cells (Fig. S6A). The results showed that, compared to KG1a cells, FTO overexpression significantly inhibited cell apoptosis, promoted cell proliferation, and caused cell cycle arrest at the G0/G1 phase. However, silencing SOX4 expression increased apoptosis (Fig. S6B), reduced proliferation (Fig. S6C), and attenuated the cell cycle arrest effect (Fig. S6D). We then found that the m6A level of the S173A mutation in FTO was lower than that of overexpressed FTO (Fig. 6B). To investigate whether O-GlcNAcylated FTO regulates the stability of SOX4 transcripts, mRNA stability analyses were performed in KG1a-FTO-HA and KG1a-FTO-S173A-HA cells. It was found that the decay rate of SOX4 mRNA was higher in KG1a-FTO-HA cells than in KG1a-FTO-S173A-HA cells (Fig. 6C). In order to explore the mechanism by which reduced O-GlcNAcylated FTO regulates SOX4 expression, KG1a-FTO-HA and KG1a-FTO-S173A-HA cellular RNA were extracted, reverse transcribed to form cDNA, and verified the SOX4 mRNA level by RT-qPCR, respectively. The results showed that the SOX4 mRNA expression level was reduced in KG1a-FTO-S173A-HA compared with KG1a-FTO-HA (Fig. 6D). SOX4 is associated with PI3K/AKT and p42-44 MAPK signaling in leukemia [32]. Our results showed that when FTO expression was increased, SOX4 expression was decreased, AKT and ERK phosphorylation was inhibited, cell survival protein Bcl2 expression was decreased and apoptotic protein Bax expression was increased in KG1a-FTO-S173A-HA (Fig. 6E). Collectively, the above data indicate that the S173A mutation in FTO increased the expression of FTO, which attenuated the stability of SOX4 transcripts in an m6A-dependent manner. Reduced SOX4 expression inhibits the activation of the PI3K-AKT and p42-44 MAPK signaling pathways and promotes apoptosis in MDS/AML cells.

## Discussion

m6A modification has been demonstrated to play a crucial role in influencing cell fate decisions during early hematopoiesis, and maintaining the identity and symmetric commitment of hematopoietic stem cells [33, 34]. Disrupting various components of the m6A machinery can shift the equilibrium between self-renewal and differentiation of healthy hematopoietic stem cells [35]. The dynamics of m6A modification correlate with cell fate decisions and differentiation trajectories of HSPCs. Most m6A modifications during hematopoiesis arise early and are critical for establishing hematopoietic cellular states [34]. Accumulated evidence shows that the m6A modification has significant effects on both normal and malignant blood cell formation, mainly by controlling the stability and efficiency of mRNA translation after transcription [36]. Moreover, it was found that m6A can impact ILF3 mRNA stability, thus regulating the advancement of multiple myeloma [37]. m6A RNA modification is a crucial regulator of hematopoiesis, playing a significant role in controlling key steps in the hematopoietic process and providing new insights for hematological tumor therapy.

Recent studies have highlighted the interplay between m6A RNA modification and post-translational modifications (PTMs). Various PTMs have been shown to regulate m6A-associated proteins and their downstream effects. For instance, the lactylation of METTL3 is critical for its ability to capture target RNA, thereby promoting the immunosuppression of tumor-infiltrating myeloid cells [38]. Reactive oxygen species (ROS) protect cells from DNA damage and apoptosis by activating the ERK/JNK signaling pathway and inducing ALKBH5 phosphorylation and SUMOylation [39]. Additionally, O-GlcNAcylation of YTHDF2 increases significantly following HBV infection [40], while O-GlcNAc modification promotes YTHDF1 and YTHDF3 activity by regulating their translation [41]. Using cell lines and conditional knockout mice (Mx1-Cre; OGT^fl/fl^), we found that O-GlcNAcylation is positively associated with m6A modification in HSCs. Although Murakami et al. reported that complete deletion of Ogt leads to rapid HSC loss [42], our experiments revealed that OGT deletion in the Mx1-Cre system was incomplete, resulting in relatively milder effects on HSCs. Interestingly, we observed a negative correlation between O-GlcNAcylation and FTO protein expression in both in vitro and in vivo models. Functional analyses revealed that O-GlcNAcylation regulates FTO protein levels by affecting protein stability rather than FTO mRNA expression. Mass spectrometry and immunoprecipitation experiments identified position 173 of the FTO protein as a critical regulatory site for O-GlcNAcylation. Substitution of serine with alanine at this site led to decreased cell proliferation, increased apoptosis, and reduced expression of SOX4, a downstream target gene of FTO. These findings suggest that O-GlcNAcylation directly affects FTO activity, thereby regulating m6A-mediated proto-oncogene expression and RNA stability.

Our study provides new evidence for the complex role of m6A modifications in RNA stability control and highlights the importance of O-GlcNAcylation as a regulatory mechanism in this process. In summary, our study demonstrates the effect of O-GlcNAcylation on FTO in MDS/AML. Specifically, we have shown that O-GlcNAcylation decreases FTO protein stability, so that low O-GlcNAcylation FTO proteins bind more SOX4, resulting in lower SOX4 m6A levels and reduced SOX4 expression. Furthermore, low levels of SOX4 prevent activation of PI3K-AKT and p44/42 MAPK pathways and enhance apoptosis. Accordingly inhibiting AML progression by OSMI-1, an OGT inhibitor, may become a new therapeutic strategy.

## Electronic supplementary material

Below is the link to the electronic supplementary material.

Supplementary Material 1

## Data Availability

No datasets were generated or analysed during the current study.
