# Supplementary material for: O-GlcNAcylated FTO promotes m6A modification of SOX4 to enhance MDS/AML cell proliferation
Source: Cell Commun Signal. 2025 Jan 23;23:43. doi: 10.1186/s12964-025-02058-6 (PMC11761745; doi:10.1186/s12964-025-02058-6)
Supplement: Supplementary file 1 — Supplementary Material 1 [file 12964_2025_2058_MOESM1_ESM.docx]

**Supplemental information**

**Materials and methods**

**RNA extraction from cultured cells and reverse transcription-quantitative PCR**

Total RNA was extracted using Trizol reagent (Thermo Fisher Scientific) and reverse transcribed into cDNA using the Superscript RT kit (Abclonal) following the manufacturer's instructions. Quantitative PCR was conducted in Bio-Rad CFX manager 3.1 with the SYBR Green Master Mix Kit (Bio-Rad). The quantification of the target genes was normalized to the quantification of the endogenous control GAPDH.

**Enzyme‑linked immunosorbent assay (ELISA)**

MDS/AML patient monocyte lysate (10 µg/mL) was dispensed into each well of a microtiter plate and incubated overnight at 4 °C. The coating solution was then aspirated and the plate was washed three times with 200 μL of 0.05% Tween 20 in PBS. Subsequently, anti-OGT antibody (ABclonal) and anti-O-GlcNAc antibody (PTM BIO, Hangzhou, China), were added and incubated at 4℃ overnight. After incubation with HRP-conjugated secondary antibody (Beyotime) at 37℃ for 1 h, 100 μL of tetramethylbenzidine (Beyotime) was added, and the color reaction's intensity was measured at 450 nm using a plate reader (Bio-Rad Laboratories, Hercules, CA, USA). All information regarding MDS/AML patients is presented in Table 1.

**Confocal microscopy analysis.**

Cells were inoculated onto confocal microscope plates (Solarbio Science & Technology; Beijing, China). Cells were cultured overnight. They were washed with PBS, fixed with 4% paraformaldehyde for 20 min and permeabilized with 0.2% Triton X-100 in PBS for 30 min. Cells were blocked with 3% BSA in PBS for 2 h and incubated with FTO primary antibody (Proteintech) overnight. Nuclei and cytoskeleton were stained with DAPI and phosphatidylinositol (Abcam; Cambridge, UK) for 15 min, respectively. Fluorescence images were captured using a confocal microscope (model TCS SP8; Leica; Mannheim, UK).


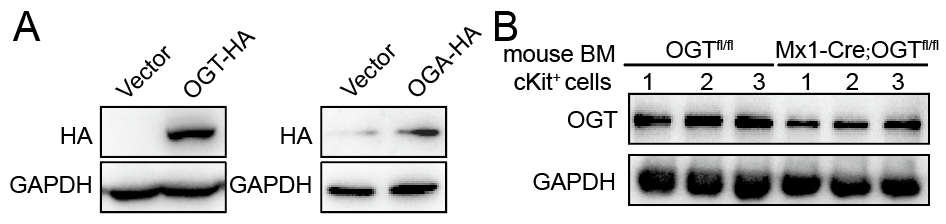


**Supplemental Fig1** (A) Western blotting shows the expression levels of OGT-HA and OGA-HA in KG1a cells. (B) Western blotting shows the expression levels of OGT in bone marrow cKit^+^ cells from OGT^fl/fl^ and Mx1-Cre; OGT^fl/fl^ mice.


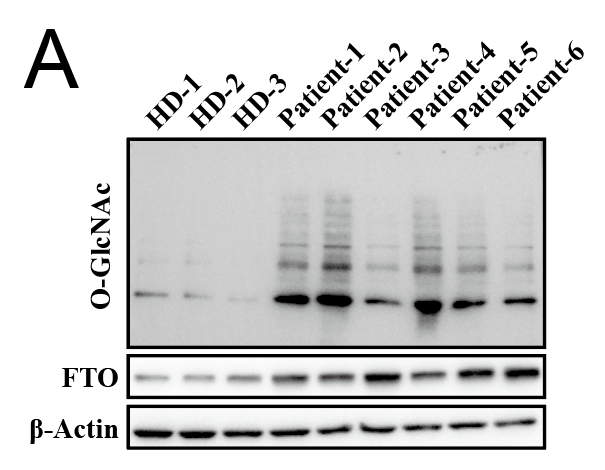


**Supplemental Fig2 (A):** Western blotting analysis showing the representative expression levels of FTO and O-GlcNAc from 6 patients and healthy donors. HD: Healthy donor.


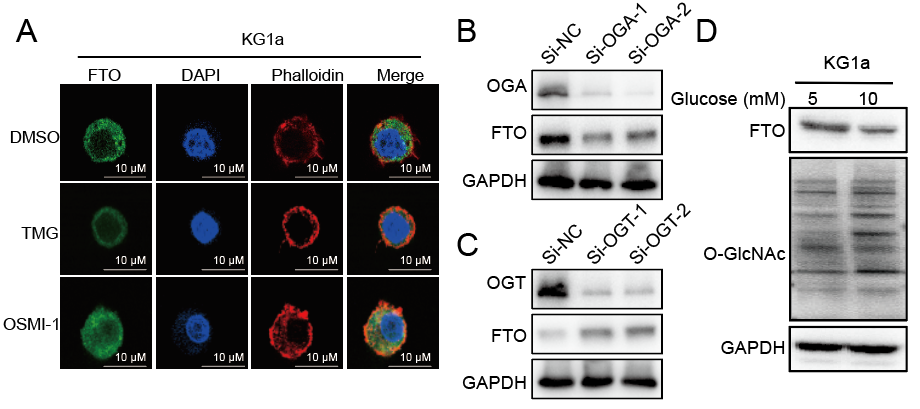


**Supplemental Fig3** (A) Confocal microscopy images showing FTO expression in KG1a cells treated with 20 μM DMSO, TMG and 20 μg/mL OSMI-1. Scale bar: 10 µm. (B). Effect of transient transfection of Si-OGA on FTO expression in KG1a cells. (C) Effect of transient transfection of Si-OGT on FTO expression in KG1a cells. (D) Effect of different concentrations of glucose on O-GlcNAc and FTO expression in KG1a.


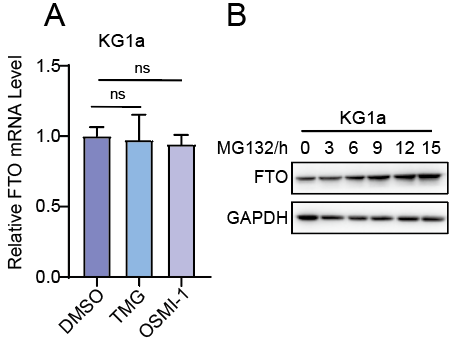


**Supplemental Fig4** (A) FTO mRNA level in KG1a cells after treatment with 20 μM DMSO, TMG and 20 μg/ml OSMI-1. (B) FTO level in KG1a after treatment with 5 μM MG132 for 0, 3, 6, 9, 12 and 15 h.


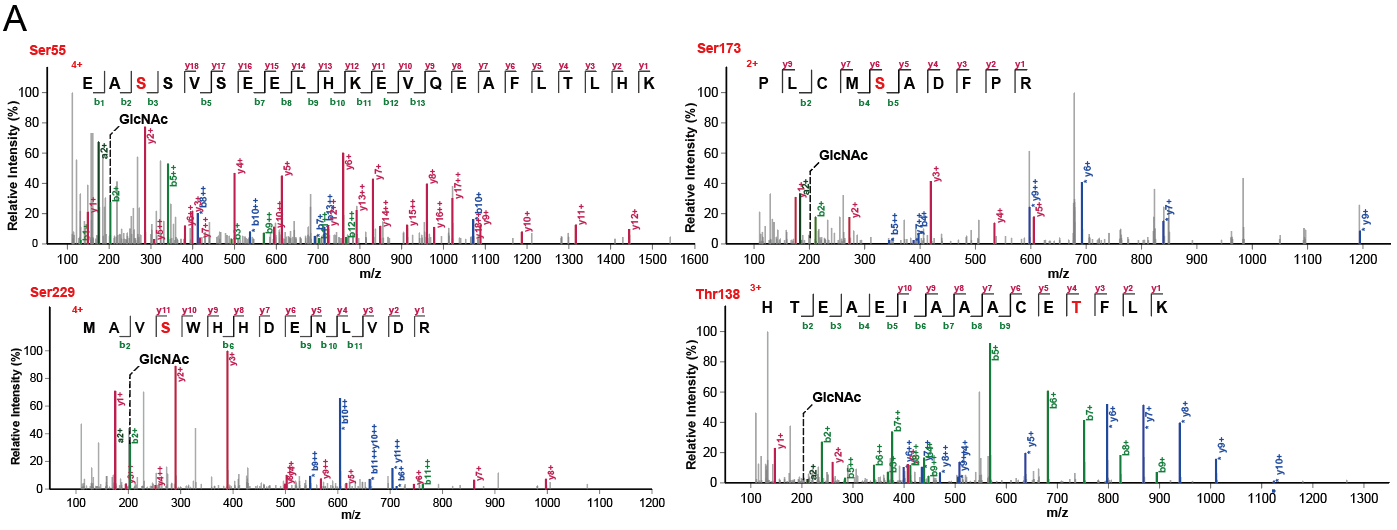


**Supplemental Fig5** (A) LC-MS analysis identified Ser55, Thr138, Ser173, and Ser229 residues on the FTO protein as O-GlcNAcylation sites. The blue chromatographic peaks correspond to the signals of O-GlcNAc-modified peptides.


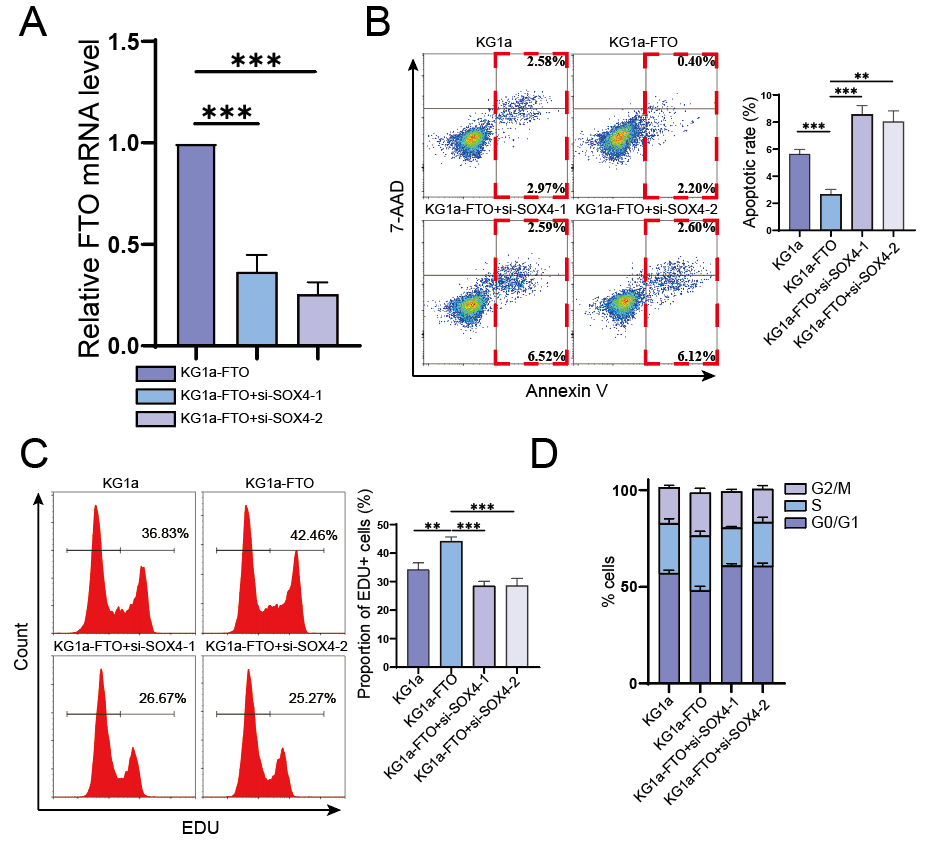


**Supplemental Fig6** (A) RT-qPCR to detect the mRNA expression levels of SOX4. (B) Cell apoptosis of KG1a, KG1a-FTO, KG1a-FTO + siSOX4-1 and KG1a-FTO + siSOX4-2 cells. (C) Cell proliferation of KG1a, KG1a-FTO, KG1a-FTO + siSOX4-1 and KG1a-FTO + siSOX4-2 cells. (D) Cell cycle analysis of KG1a, KG1a-FTO, KG1a-FTO + siSOX4-1 and KG1a-FTO + siSOX4-2 cells were conducted using flow cytometry.

**Table 1**

**Information of MDS/AML patients**

| **Diagnosis** | **Age (year)** | **Gender** | **Subtype** |
| --- | --- | --- | --- |
| HD | 65 | M |  |
| HD | 34 | M |  |
| HD | 51 | F |  |
| MDS  MDS  MDS  MDS  MDS  MDS  MDS  AML  AML  AML  AML  AML  AML  AML | 71  46  66  57  56  73  59  85  38  74  56  57  34  76 | F  M  F  M  F  M  M  F  M  M  F  M  F  F | RAEB  RA  RAEB-t  RARS  RAEB  RA  RAEB-t  M3  PML/RARA L  CBFB/MYH11  RUNX1/RUNX1T1  PML/RARA L  M3  CBFB/MYH11 |
